# Examining the pedagogical practices that support cultural proficiency development in graduate health science students

**DOI:** 10.1186/s12909-024-05097-8

**Published:** 2024-02-09

**Authors:** Julie E. Speer, Quincy Conley

**Affiliations:** Teaching & Learning Center, A.T. Still University, 5835 E. Still Circle, Mesa, AZ 85206 USA

**Keywords:** Bias, Empathy, Inclusion, Instructional design, Osteopathic health sciences

## Abstract

**Background:**

Health disparities are often a function of systemic discrimination and healthcare providers’ biases. In recognition of this, health science programs have begun to offer training to foster cultural proficiency (CP) in future professionals. However, there is not yet consensus about the best ways to integrate CP into didactic and clinical education, and little is known about the role of clinical rotations in fostering CP.

**Methods:**

Here, a mixed-methods approach was used to survey students (*n* = 131) from a private all-graduate level osteopathic health sciences university to gain insight into the training approaches students encountered related to CP and how these may vary as a function of academic progression. The research survey included instruments designed to quantify students’ implicit associations, beliefs, and experiences related to the CP training they encountered through the use of validated instruments, including Implicit Association Tests and the Ethnocultural Empathy Inventory, and custom-designed questions.

**Results:**

The data revealed that most students (73%) had received CP training during graduate school which primarily occurred via discussions, lectures, and readings; however, the duration and students’ perception of the training varied substantially (e.g., training range = 1–100 hours). In addition, while students largely indicated that they valued CP and sought to provide empathetic care to their patients, they also expressed personal understandings of CP that often fell short of advocacy and addressing personal and societal biases. The results further suggested that clinical rotations may help students attenuate implicit biases but did not appear to be synergistic with pre-clinical courses in fostering other CP knowledge, skills, and attitudes.

**Conclusions:**

These findings highlight the need to utilize evidence-based pedagogical practices to design intentional, integrated, and holistic CP training throughout health science programs that employ an intersectional lens and empowers learners to serve as advocates for their patients and address systemic challenges.

**Supplementary Information:**

The online version contains supplementary material available at 10.1186/s12909-024-05097-8.

## Background

Disparities in health and healthcare access globally exist based on intersecting aspects of a patient’s identity (including, but not limited to, race, ethnicity, religion, language, socioeconomic status, citizenship/immigration status, physical and mental health and wellbeing, gender expression, and sexual identity) [1–6]. These disparities are a function of a long history of systemic discrimination. However, implicit and explicit biases of individual care providers can propagate these inequities, which can manifest as altered treatment planning, adverse outcomes, or increased morbidity and mortality rates for patients with one or more minoritized identities [7–12]. In response to this reality, many health disciplines have been working to incorporate opportunities for trainees to engage with lessons on topics related to diversity, equity, inclusion, and justice (DEIJ), such as communicating on sensitive topics, leading with empathy, recognizing systemic bias, seeing culture as dynamic and multidimensional, or recognizing one’s own cultural identities and valuing those of others. Regarding the latter, several terms are used throughout the literature and in practice to describe the discourse on this concept, such as cultural proficiency (CP), cultural competence, and cultural humility. Though the origins and nuances of these terms can vary, all three highlight several key factors – the importance of valuing that others have life experiences that are different than one’s own, the imperative of working to advocate for equity, and the necessity of continuous self-reflection and learning [13–17].

CP is often seen as a concept that incorporates cultural competence and humility and can be used as a model for individuals, institutions, and systems [16, 17]. For example, in their guide on culturally proficient instruction [18], Nuri-Robins and colleagues write about CP as follows:

“The policies and practices of an organization or the values and behaviors of an individual that enable the organization or person to interact effectively in a culturally diverse environment; reflected in the way an organization treats its employees, its clients, and its community; an inside-out approach to issues arising from diversity; a focus on learning about oneself and recognizing how one’s culture and one’s identity may affect others, not on learning about others.” (p. 56).

**Fig. 1:**
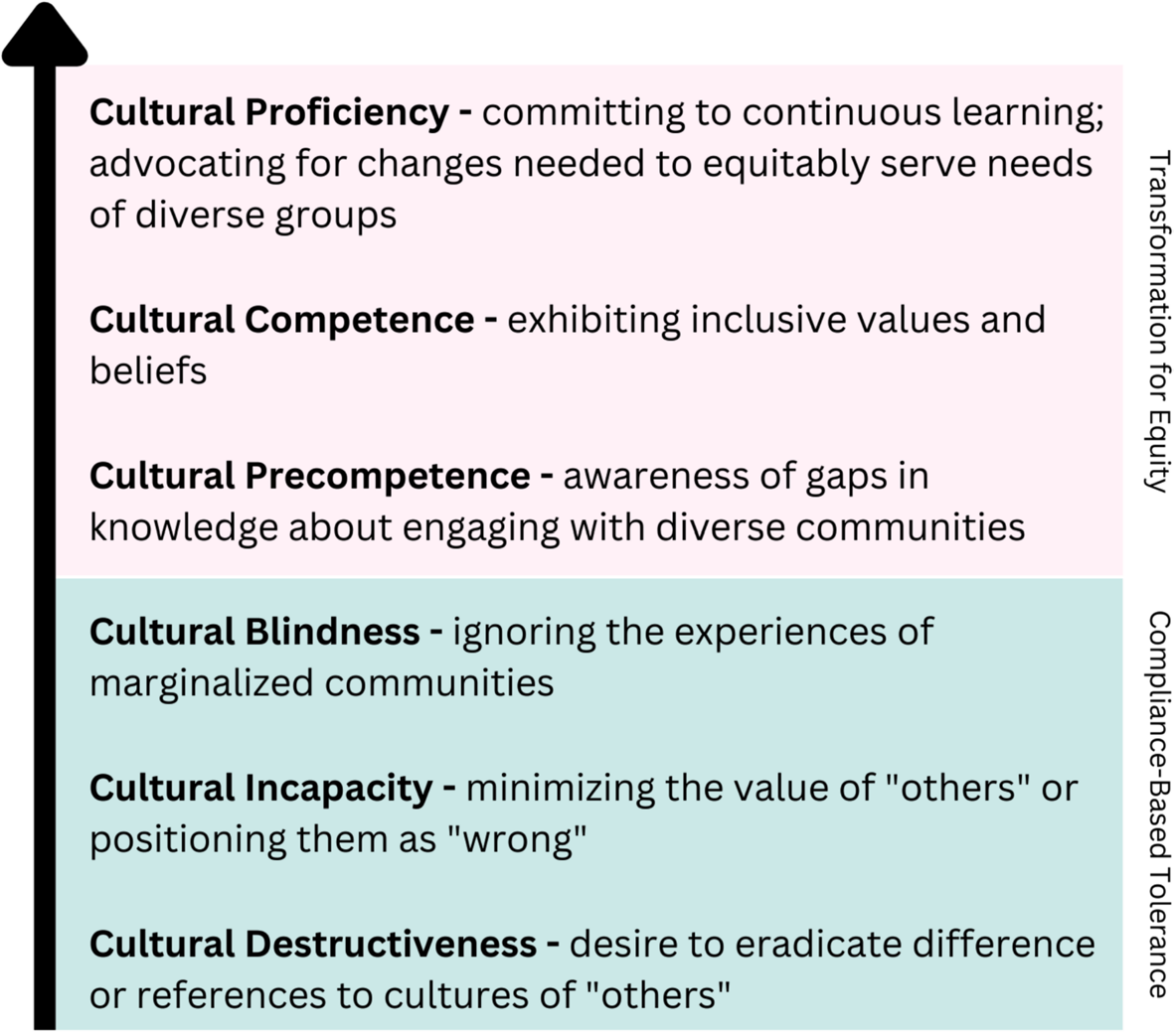
Overview of the Continuum of Cultural Proficiency [16, 18]

This framework posits there are five central factors of CP: assessing cultural knowledge (naming the differences), valuing diversity (claiming the differences), managing dynamics of difference (reframing the differences), adapting to diversity (training about differences), and institutionalizing cultural knowledge (changing for differences) [16, 18]. Integrating these factors effectively into every aspect of practice is the ultimate goal; however, organizations and the individuals that comprise them may be located (and dynamically shift) along a spectrum or a continuum ranging from cultural destructiveness to cultural proficiency (Fig. 1) [16, 18].

The first half of the continuum reflects an approach that centers on compliance-based tolerance for diversity [16, 18]. In this realm, an organization or individual might exhibit cultural destructiveness, cultural incapacity, or cultural blindness. While these each describe a different point along the continuum, together they indicate a tendency to minimize the needs of diverse communities by ignoring different life experiences [16, 18]. In contrast, the second half of the continuum seeks to drive transformation for equity – albeit to varying degrees [16, 18]. Cultural precompetence reflects becoming aware of gaps in one’s knowledge about engaging with individuals from marginalized communities [16, 18]. Cultural competence and proficiency move beyond this “recognition” stage by leaning into proactive behaviors [16, 18] and though “proficiency” may suggest the end of a journey, here this term implies a commitment to continuous learning and embracing advocacy and allyship [16, 18].

Utilizing these skills is vital in daily life as community members and colleagues and is essential for providing affirming, empowering, patient-centered care and cultivating positive and inclusive learning environments. However, there is not yet consensus about the most effective instructional approaches for promoting a shift towards cultural competency and proficiency amongst individuals and across disciplines. A body of literature suggests that despite best intentions, DEIJ and CP training can fail to promote long-term changes in beliefs and behavior for various (often interrelated) reasons [19–22]. One challenge is using standalone or “one-off” DEIJ or CP trainings such as a single lectures or annual trainings [23, 24]. ﻿One meta-analysis found that standalone DEIJ trainings had a significantly lower effect size than trainings which leveraged a more integrated approach [23]. The difference in impact can be due to several causes, including the scope and duration of the training and de-contextualization of the information. But as the authors of the meta-analysis suggest, standalone trainings may also have a “check-off-the-box” feel that focuses more on compliance rather than demonstrating a top-down organizational commitment to learning and cultural change, which in turn can impact participants’ motivation and commitment to the learning [23]. Another factor that can limit long-term effects is if trainings take an overly simplistic approach. While oversimplifications can present differently, prior literature has indicated several common “pitfalls” including trainings that touch on aspects of diversity individually rather than addressing intersectionality, instructional methods that focus purely on cognitive learning goals without incorporating critical reflection, utilization of an “us-versus-them” approach, and those that present cultural or identity groups as static and uniform [21, 25–27]. Additionally, many trainings and DEIJ efforts have been shown to unintentionally reinforce rather than challenge and mitigate stereotypes [21, 25, 28]. This can occur because asking people to suppress stereotypes may fortify them by making them cognitively more accessible [21, 24]. Furthermore, while incorporating diverse lived experiences into case studies or classroom examples is important and can be well-intended, it can also inadvertently activate and reinforce stereotypes and be triggering or traumatizing when done without careful examination or when marginalized voices are not authentically centered [21, 29–32].

Such reports on ineffectual training methods should not be taken to suggest that impactful DEIJ and CP training can’t exist. On the contrary, these data highlight the value of drawing from student-centered pedagogical theories when developing training related to CP to promote training transfer and long-term retention, as well as the need to utilize educational research methods to determine the efficacy and perceptions of the intervention.

One student-centered educational approach commonly used across disciplines is experiential learning [33]. Osteopathic and allopathic health science programs have long employed this “learn-by-doing” strategy which seeks to integrate classroom and community-based education into the curriculum to accelerate students’ on-the-job readiness upon graduation [34]. Additionally, graduate programs may incorporate opportunities for students to conduct extended rotations at community health clinics (CHCs) in underserved communities around the country [33, 35–37]. This movement towards embedded learning experiences in local communities aligns with the health profession’s desire to prepare emerging clinicians with skills and expertise to address health disparities. But it is crucial to understand the approaches to, and impact of, classroom learning and clinical education in CHCs on students’ learning outcomes related to CP. Furthermore, there is an opportunity to determine which interventions and experiences are most impactful in promoting the development of skills, knowledge, and attitudes associated with recognizing and mitigating bias, promoting and practicing empathetic and equitable healthcare practices, and developing cultural proficiency.

Therefore, the present study sought to employ a mixed-methods educational research approach based on the framework discussed by Nuri-Robins et al. in order to catalogue the CP training experiences students across an osteopathic health sciences institution encountered and to explore differences in students’ CP beliefs and skills based on participation in community-based education. This approach sought to leverage previously developed and validated instruments for measuring participants’ values, beliefs, and automatic associations, as well as custom survey questions that provided opportunities for students to detail their experiences and perceptions of the training experiences they encountered and to describe their own understanding of CP.

## Methods

### Recruitment and participants

Students at a medium-sized osteopathic graduate-level health science university were invited to participate in the IRB-approved survey (IRB# 2022–092). The institution has an approximate enrollment of 3800 students and has three campus locations in the United States (Missouri, Arizona, and California). Although depending on the academic program, students may participate in training remotely or through rotations at clinical sites, the number, duration, and locations of which vary by program (Additional File 1). Invitations to participate in the survey were disseminated through on-campus message boards and distributed to students via listservs managed by program administrators and faculty. The announcement contained a brief description of the project with a link or a QR code to access the informed consent materials, which had additional details regarding the purpose of the study, eligibility criteria (18+ years of age and currently enrolled at the institution), potential risks and benefits, and compensation. These materials also indicated that the study was being conducted by university staff and not by faculty members, that compensation for participating was a raffle to win one of 30 $50 gift cards (a benefit unrelated to coursework or grades), and that students could access the survey over an approximately one-month period. Students who chose to enroll (by providing informed consent) were allowed to continue to the online survey (Qualtrics; Provo, UT). Demographic information for the 131 students who completed the survey can be seen in Table 1.

**Table 1:** Demographic information for survey participants

Demographic Factors	*n* (%)
Age	
Average	31.33
Range	22–63
Standard deviation	10.39
Current Gender Identity^a^	
Woman	97 (74.62)
Man	30 (23.08)
Gender Queer, Questioning, Other, or preferred not to answer	3 (2.31)
Ethnicity and Race	
American Indian/Alaskan Native	1 (0.73)
Black or African American	9 (6.57)
East Asian	8 (5.84)
Middle Eastern	2 (1.46)
Multiracial or Unknown	15 (10.95)
Native Hawaiian or Pacific Islander	1 (0.73)
South Asian	6 (4.38)
White	95 (69.34)
Hispanic or Latinx	10 (7.81)
Not Hispanic or Latinx	114 (89.06)
Unknown or preferred not to answer	4 (3.13)
Education Program	
Clinical Allied Health^b^	60 (44.44)
Dental School	19 (14.07)
Graduate Health Studies^c^	34 (25.19)
Medical School	21 (15.56)
Other	1 (0.74)
Primary Campus Location of Participant’s School/College	
Missouri	29 (21.6)
Arizona	71 (53.0)
California	0 (0)
Remote	34 (25.4)
Degree in Progress	
Doctorate	121 (90.30)
Masters	13 (9.70)
Approximate Program Completion (%)	
Average	60.81
Standard Deviation	30.50
Rotation Completion	
Hasn’t yet completed a rotation, or in a program that doesn’t complete rotations	62 (47.33)
Currently in a rotation	38 (29.01)
Completed one or more rotation(s) but not currently rotating	31 (23.66)
Average number of rotations completed	4.40
Standard deviation	4.45

^a^The survey question was open ended and asked students to state their current gender identity

^b^Clinical allied health programs include athletic training, physical therapy, occupational therapy, physician assistant, and audiology

^c^Graduate health programs include public health, health administration, and education in health professions

### Research survey

#### Overview

After providing consent, participants were immediately given access to the research survey, which was designed to take between 20 and 30 minutes to complete. The survey was broken up into blocks based on the research instruments (described in detail below), and specific instructions were provided at the beginning of each block. Given the sensitive nature of this topic, participants were only required to engage with the implicit association tests (IATs) and answer several questions, one about what type of device they were taking the survey on (which was needed to provide the implicit association tests with proper functionality), one that asked them about whether they had started or completed any clinical rotations (which was used to bin the results as a proxy for academic progression), and one question which was required to support the logic of the survey (i.e., where an answer triggered a subset of questions to appear). Otherwise, participants were alerted if they left questions blank but were permitted to move forward without answering if they chose to do so. At any time, students could close the survey without submitting. After completing the survey, participants were presented with a message that thanked them for their time and provided a link to a separate Qualtrics survey where they could record their email address if they wished to be entered into a raffle to win a gift card (an incentive for participation). The use of these two independent surveys was required to retain the anonymity of responses in the research survey.

As discussed further in the sub-sections below, the survey instruments were designed to leverage both custom-written and previously validated instruments to assess students’ understandings and experiences, subconscious associations and implicit reactions, as well as their conscious beliefs about themselves and their actions. A multitude of instruments have been used in the literature and extensively reviewed elsewhere that are designed to capture this data [38–40]. Each tool has its own pros and cons and particular scope, such as assessing self-perception, cultural knowledge, program-level outcomes, and patient-provider interactions. Together, the research team, consisting of experts in medical education and educational research methodology with extensive experience in survey design and adaptation, reviewed instruments for their applicability in this study based on their collective potential to capture data at multiple levels (namely, both implicit reactions and self-evaluation). Additionally, the instruments were considered according to the following criteria: the instrument must be able to be administered via an online survey, the instrument and its questions or files must be fully publicly available and can be used free of cost, the instrument must be applicable across healthcare disciplines (e.g., not specific to nursing, dentistry, medicine, etc.), and the instrument must allow data to be captured in a time-effective manner (a total survey length of approximately 20–30 minutes was targeted given how busy students are and to prevent attrition due to survey length). Once these criteria were met, and before their inclusion in the survey, the selected instruments underwent further review, guided by best practices and the collective expertise of the research team. This iterative process aimed to ensure that the validated instruments were tailored to the specific nuances of this study context and objectives and aligned with survey research standards in medical education.

#### Semi-quantitative and qualitative questions

Participants were asked to respond to Likert-type and open-ended questions (Additional File 2) about their understanding and knowledge related to CP (e.g., “What does it mean to you for someone to exhibit cultural proficiency?”), the amount and types of CP training they have received (e.g., “How has instruction and practice in cultural proficiency been incorporated into your training?”, “Please estimate the number of hours of cultural awareness, bias mitigation, and/or cultural proficiency training…that you have received…”), and their demographic information (Table 1). The language in these questions was informed by prior surveys conducted by the authors and those used broadly in health science education [7, 41–46]. Both authors contributed to the drafting and refining of these questions. Additionally, the language used in these questions was chosen to reflect terminology that is common in this area of study or is frequently used within the parlance of the institution where the study was conducted. However, in several instances, intentionally subjective language (e.g., “eye-opening experiences”) was used so as not to bias the responses of students and to capture participants’ authentic perspectives based on the experiences that resonated with them.

While every effort was taken to analyze each student’s responses, there were several questions where data was provided in a way that couldn’t be interpreted accurately by the research team and, therefore, was excluded from the data set. For example, regarding the questions related to duration of training, answers that were not provided in units of hours were not analyzed (e.g., “Took a 3 credit hour class in undergrad”) because the data couldn’t accurately be converted to hours of instruction without additional information. If students provided a range of hours (e.g., “12–24…”) the average of the range was used. In contrast, if students provided a response like “10+ hours”, the minimum number of the provided inequality was used.

The qualitative data were analyzed by one author (JS) using ATLAS.ti (ATLAS.ti Scientific Software Development GmbH; Berlin, Germany) to identify and semi-quantify emergent themes amongst the responses [47, 48]. Both authors reviewed the thematic codes and resulting data, and any discrepancies were discussed and resolved as a research team.

#### Implicit association tests

**Fig. 2:**
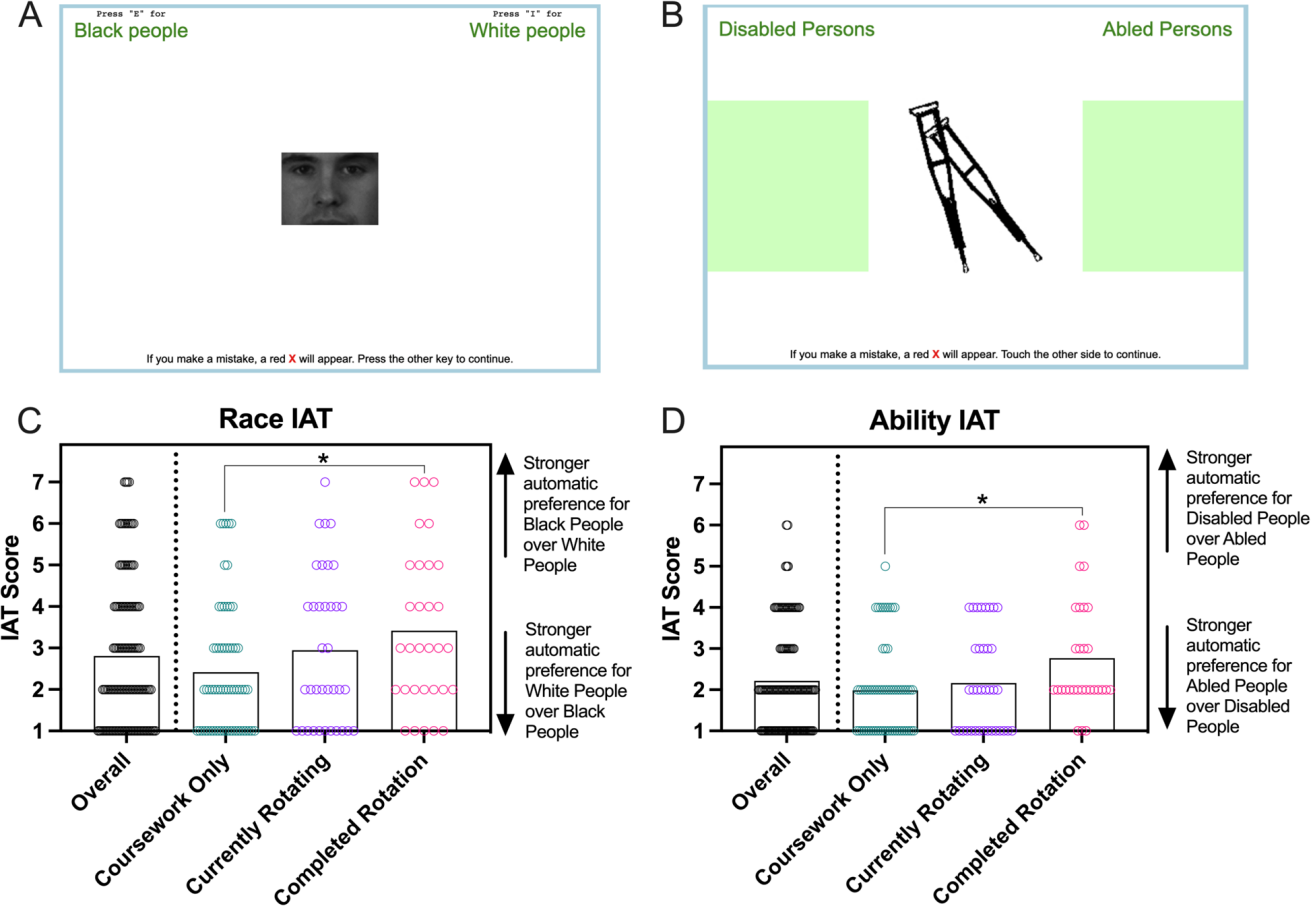
Implicit Association Tests (IATs) were encoded in the Qualtrics-based research survey that enabled respondents to complete the tasks using a keyboard (**A**) or touchscreen (**B**). Results from the IATs that explored associations based on race (**C**) and physical ability/disability (**D**) ranged from 1 to 7. The height of each bar = mean for that group; each dot = data from 1 respondent; * *p* < 0.05

Implicit Association Tests (IATs) are instruments that have been widely used across disciplines, including healthcare, to measure differences in individuals’ subconscious associations between concepts (e.g., race, ability, gender) and evaluations or stereotypes (e.g., good or bad, safe or dangerous) [49–51]. During these online tests, participants are prompted to perform categorization tasks as quickly as possible [49, 50]. They are asked to press one button if the object or word they see belongs to one category and another button if it belongs to the other (Fig. 2A-B) [49, 50]. In early rounds of the test (blocks), participants are prompted to categorize single objects or words [49, 50]. For example, in the first part of the Race IAT, a participant would be presented with a picture of a face and asked to decide if that face corresponds more with the category “White Persons” or “Black Persons.” The second part asks participants to sort words into categories, so for example, if the word “Joy” appeared, the subject would be asked to determine whether that belongs in the “Good” or “Bad” category [49, 50]. In later blocks of the test, categories are combined (e.g., Black Persons/Good or White Persons/Bad), and participants are tasked with determining whether a picture or word belongs to the combined Black Persons/Good category or the White Persons/Bad category [49, 50]. As the test continues, the categories are shuffled to represent the other combination of the concept and evaluation (e.g., Black Persons/Bad, White Persons/Good) [49, 50]. The order of these blocks is randomized so that some participants see the categories Black Persons/Good and White Persons/Bad first, and others will see Black Persons/Bad and White Persons/Good first [49, 50]. Additionally, the test includes blocks where the buttons for the categories are flipped [49, 50]. For example, if participants were previously told to use the “i” key to represent “Good” and the “e” key to represent “Bad,” they would now be asked to use “e” to sort an object into “Good” and “i” to sort one into “Bad.” The final IAT score, which reflects the strength of associations, is based on the time required to complete the task [49, 50]. This is because cognitive research suggests that the more strongly two concepts are associated, the more quickly they will be sorted; while concepts that are not as strongly associated will require more time to categorize [49].

In the present study, two IATs, race and disability/ability [49, 51, 52], were each embedded into the survey via the JavaScript editor in Qualtrics according to previously described methods and using published code [52–54]. These particular IATs were selected because they aligned with the research questions and study population. Within the survey, two versions of each IAT were generated in order to allow participants to take the appropriate version based on the device they were using (computer v. tablet/phone) (Fig. 2A-B) and instructions informed the participants of how to use their keyboard or touchscreen to respond to each prompt. Once students completed each IAT, the script calculated a final score (values ranging from 1 to 7) that was based on the time spent assigning each image to the respective word or phrase [52, 53]. Final scores closer to 1 indicated stronger automatic preferences for White Persons or Abled Persons, while values closer to 7 indicated stronger automatic preferences for Black Persons or Disabled Persons. While the scores were available to the authors via Qualtrics, students were not presented with the results of their IATs.

#### Ethnocultural empathy inventory

A 15-question Ethnocultural Empathy Inventory (EEI) was employed from the ﻿Cross-Cultural Competence Inventory [55] which itself was adapted from prior instruments including the Scale of Ethnocultural Empathy [56]. The EEI was chosen for the present study due to its applicability to all disciplines partaking in the study and its ability to quantify participants’ self-perceptions of their own beliefs which could serve an important function of triangulating data alongside the IATs. This instrument (Additional File 2) contains statements to which participants indicate their agreement or disagreement (scale of 1–6; 1 = strongly disagree, 6 = strongly agree), such as, “When dealing with people of a different ethnicity or culture, understanding their viewpoint is a top priority for me” [55, 56]. A final inventory score for each participant was calculated by first applying reverse scoring to the appropriate questions [55, 56] and then summing the responses across each participant. A higher score on this instrument is typically more indicative of responses that suggest empathy and compassion towards others with different lived experiences than the respondent.

#### Data visualization and statistical analysis

Data visualizations and statistical analyses were completed using Prism v9 (GraphPad Software; San Diego, CA) unless otherwise described. Descriptive statistics, including mean and ranges, were calculated. Percentages and counts of responses were also quantified. Categorical responses to the Likert-style questions were converted to numerical values. Then, differences in the data by rotation status were assessed using Kruskal-Wallis tests for one-way analysis of variance and Dunn’s multiple comparison tests. These statistical tests were also used to determine if there were differences in the IAT and Ethnocultural Empathy Inventory results by rotational status. In addition, a one-way analysis of variance (ANOVA) was used to determine whether differences existed in training duration across the rotational groups. Finally, Cronbach’s alpha was calculated in Excel (Microsoft Corporation; Redmond, WA) to determine the internal consistency of the responses to the Ethnocultural Empathy Inventory.

## Results

### Training experiences and perceptions

**Fig. 3:**
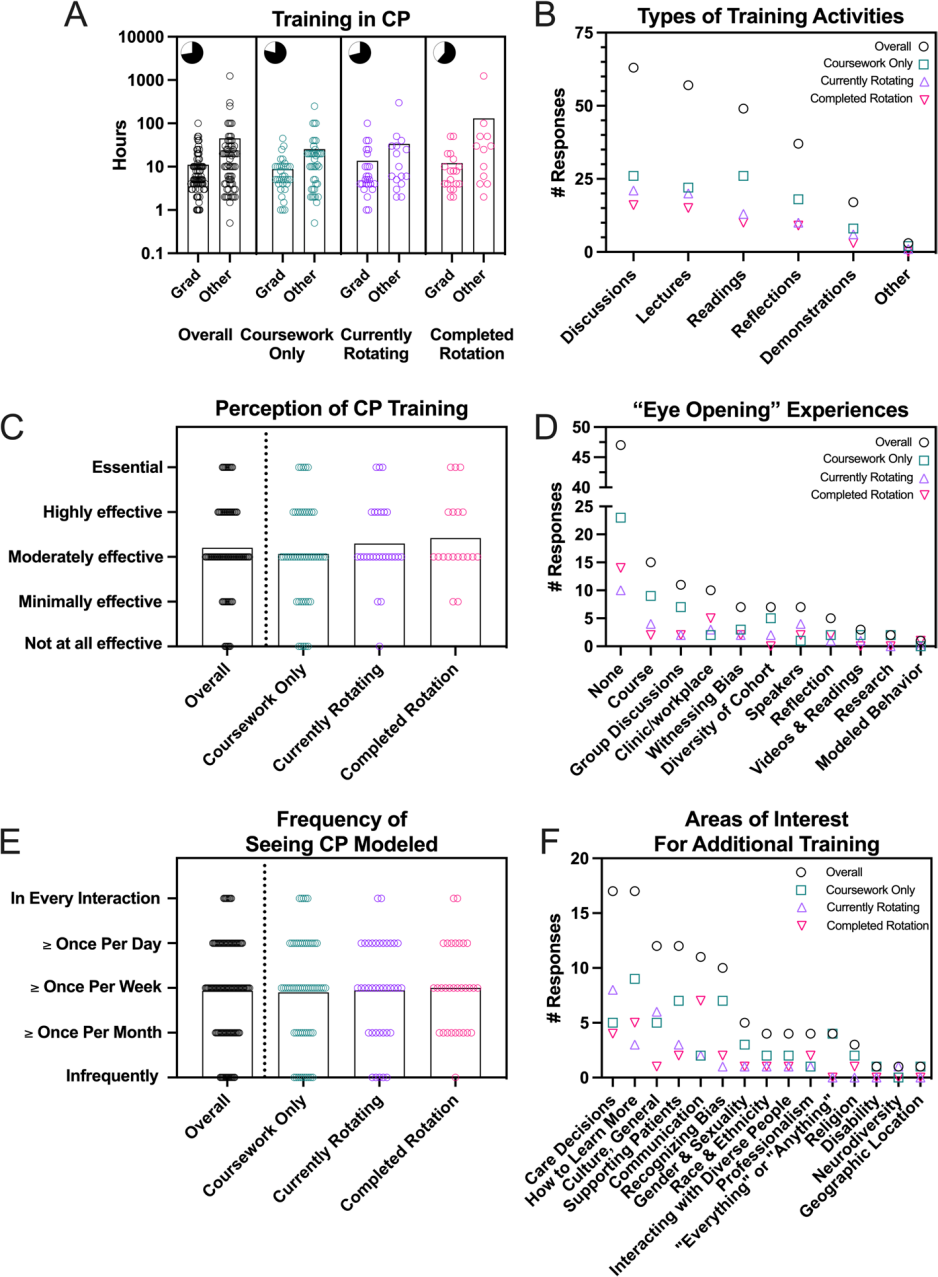
Survey questions sought to catalog students’ experiences related to the training they had received in CP. Students were first asked to indicate whether they had (black) or had not (white) undergone training (pie charts, **A**). Then, if they had received training, students provided an estimated duration of the training they engaged with during their tenure in graduate school (Grad) or through other avenues (Other). Students indicated the types of materials/activities they encountered (**B**) and their perception of the training (**C**). Students were also asked to reflect on an element of their training that was particularly “eye opening” (**D**) and to share how frequently they observe others model culturally proficient behaviors (**E**). Lastly, students were asked what CP topics they would like additional training on (**F**). For A, C, and E, bars = mean response, and each dot represents data from 1 respondent. For B, D, and F, the symbols indicate the number of responses for a given category for each group (rotation status)

The majority (73%) of respondents indicated that they had received some training in CP either as part of graduate school or outside of their current studies (e.g., during undergraduate courses, through professional organizations, or previous work experiences; Fig. 3A (pie chart inserts). However, the amount of training participants received was variable (Fig. 3A). The average duration of training received within graduate school was 11 hours (range = 1–100 hours) and was not statistically different based on rotational status (*p* = 0.44). The average amount of training received before or outside of graduate school was 45 hours, ranging from 0.50 to 1248 hours.

Respondents indicated that their training predominantly consisted of discussions, lectures, readings, and to a lesser degree, reflections, demonstrations, or other activities (Fig. 3B), and the format of training experiences was similar regardless of rotation status. On average, students rated this training as being moderately effective (Fig. 3C); however, responses varied. Some students perceived the training as less effective, while others found it to be more effective or even essential. This range in perception was also seen among responses to a question that asked students to reflect on which elements of their training were particularly “eye opening.” 47 respondents (41%) either didn’t or couldn’t identify aspects that were particularly helpful in learning skills related to appreciating differences and mitigating bias (Fig. 3D). Others, however, indicated that they learned most from experiences including course work, group discussions, encounters in the clinic or workplace, personally witnessing/experiencing bias, or seeing culturally proficient behaviors demonstrated by colleagues or mentors. Regarding the latter, a plurality of respondents (36%) indicated that they typically see CP modeled once per week (Fig. 3E). A nearly equal number of respondents indicated they see CP modeled for them less frequently (32%; once per month or infrequently) or more frequently (32%; daily or in every interaction). Responses on the frequency of seeing CP modeled appeared to be similar throughout the educational experience (*p =* 0.96).

The majority of respondents (across all rotation groups) indicated that, despite receiving some training, they would benefit from continued education related to cultural proficiency (68% of students agreed continued training would be helpful, 21% were unsure, and only 11% said no). The topics students were interested in learning more about were fairly diverse (Fig. 3F), but common responses indicated an interest in learning more about how to make care decisions in the clinic and ways to best show support to their patients. For example, one student responded that they were interested in knowing more about “[h]ow different cultures might react to certain treatment suggestions (transfusions, certain meds, etc.), [the] background behind that reaction, and how to best approach it with them.” Other common themes of interest for continued training included: communication (particularly around difficult or sensitive issues), topics related to specific cultural and identity groups (neurodiversity, gender and sexuality, disability, religion, race, and ethnicity), and knowledge and awareness of cultural groups in general.

### Knowledge of and beliefs related to cultural proficiency

**Fig. 4:**
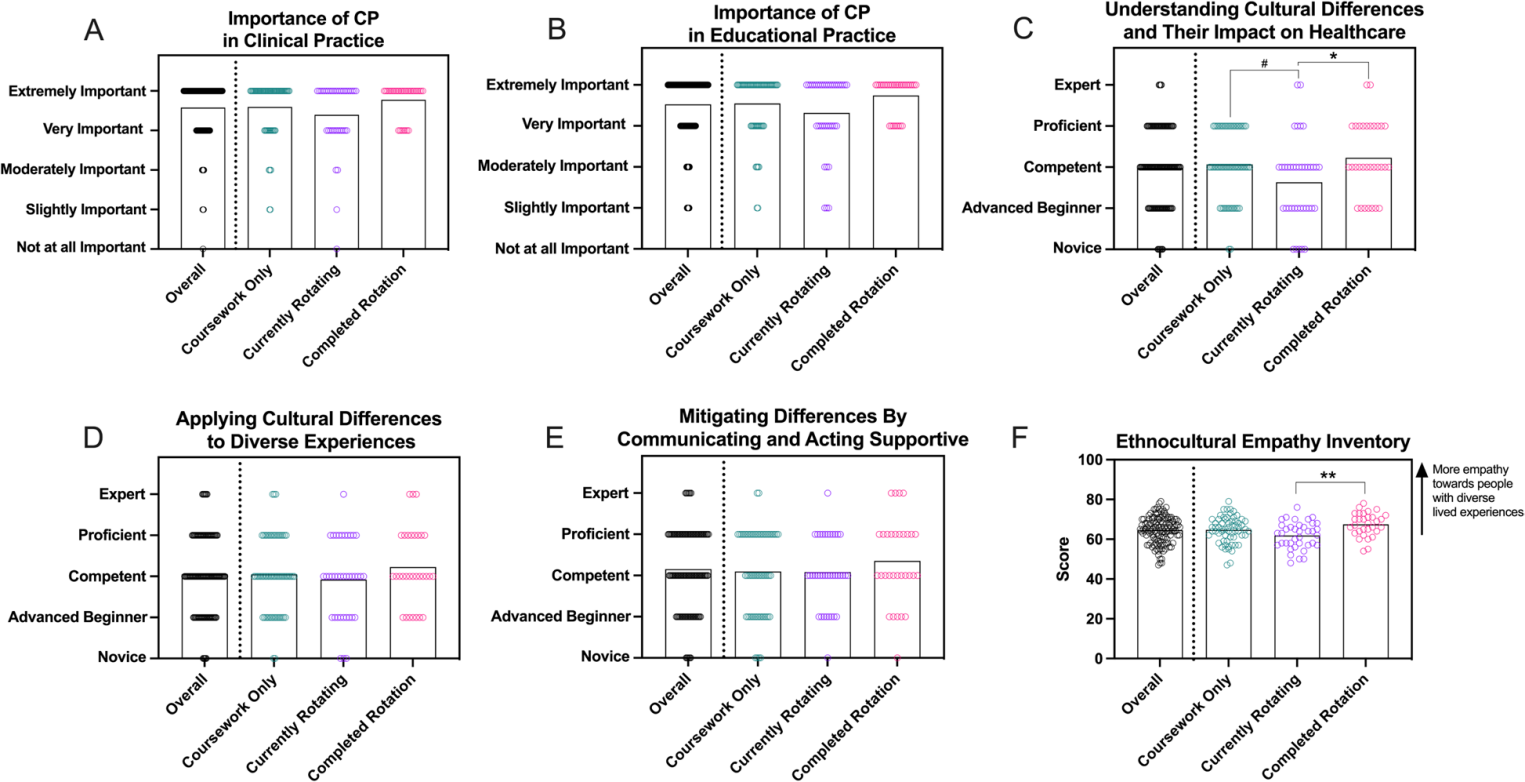
Students’ self-evaluations of knowledge, skills, and attitudes related to CP. Students indicated how important they felt CP was in clinical (**A**) and educational (**B**) contexts. They also provided self-ratings for three “meta-skills” related to CP (**C-E**) and engaged with the 15-question Ethnocultural Empathy Inventory (**F**). Bars = mean, each dot = response from 1 participant, ** *p <* 0.01, * *p* < 0.05, # *p* < 0.06

Most students (> 80% regardless of rotation status) indicated a strong understanding of how CP was important for both clinical (*p =* 0.15, Fig. 4A) and educational practices (*p =* 0.14, Fig. 4B). The majority of students also rated themselves highly (> 70% of students indicated answers between competent and expert) on three meta-skills related to CP [57] (Fig. 4C-E; How would you rate your: “understanding of the complex elements inherent to cultural differences and their impact on health and healthcare delivery,” “ability to apply an understanding of cultural differences through active participation in diverse cultural experiences and opportunities,” and “ability to mitigate differences by communicating and acting in a supportive manner and recognizing other cultural group perspectives.”). Self-ratings towards these skills were similar amongst students of different rotational statuses for the second and third meta-skill (*p =* 0.55 and 0.47, respectively). However, students who had not yet started a rotation or who had previously completed a rotation rated their abilities to understand how cultural differences impact healthcare more highly than those currently in a rotation (*p* = 0.02).

Responses to the Ethnocultural Empathy Inventory also indicated that most students valued awareness and respect for difference and an interest in supporting inclusive practices. The average overall score on the instrument was 64.5, and scores only differed significantly between students who were currently rotating and those who had completed rotations (*p <* 0.01, Fig. 4F). The average response across the 15 questions was 4.30, indicating moderate disagreement/agreement with the statements. The question with the highest average score (5.23, after reverse-scoring was applied to this question) was, “I don’t understand why people of different ethnicities or cultures feel they have to cling to their own values and traditions.” The question with the lowest average score (1.98, after reverse-scoring was applied to this question) was, “I try to look for a logical explanation or solution to almost every problem I encounter.” A Cronbach’s alpha score of 0.65 suggests moderate internal consistency and that the instrument was valid in this population [58].

**Fig. 5:**
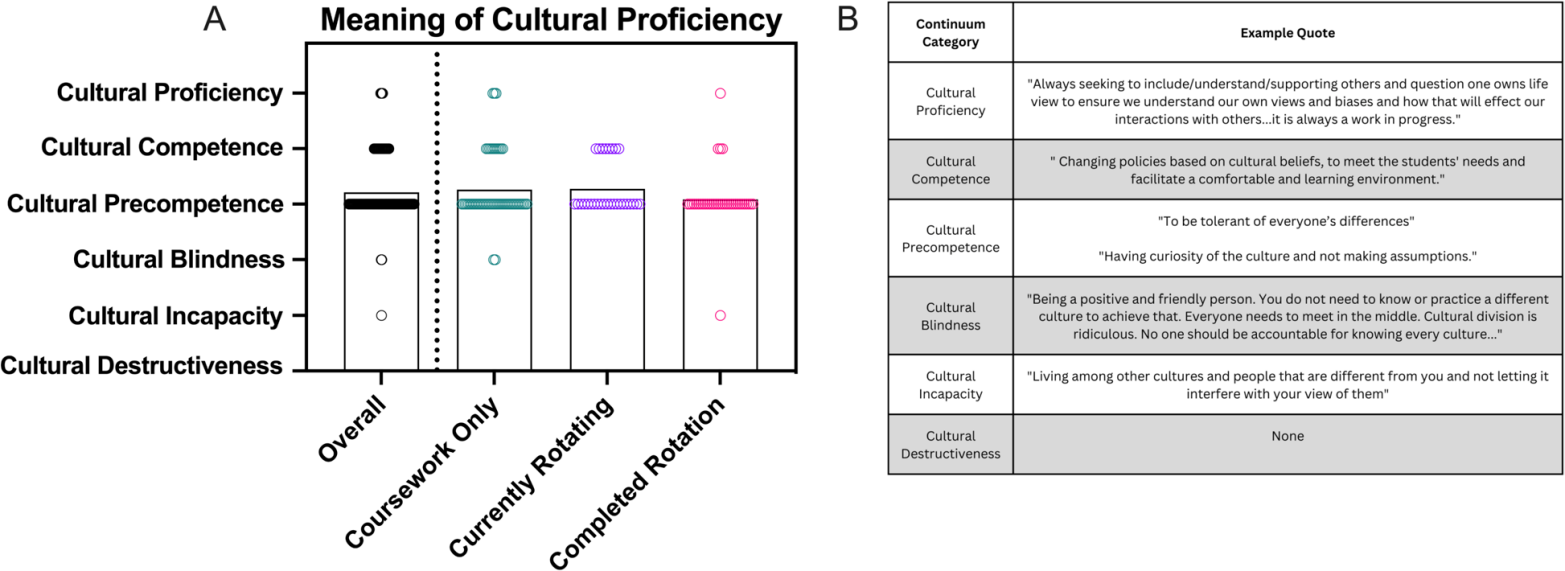
Students’ definitions of cultural proficiency were coded according to where they fell on the spectrum of cultural proficiency (**A**) with examples of each provided (**B**)

While students largely indicated they understood these concepts and expressed a desire to exhibit inclusion and empathy, personal definitions of CP varied across responses. When asked the open-ended question, “What does cultural proficiency mean to you?”, a plethora of responses included phrases like “respecting differences,” “being kind to others,” or “open-mindedness.” Others referred to the need to be “aware” of other cultures or to “understand” different experiences or traditions. These responses indicate that most (86%) students seemed to understand some components of CP (particularly those related to assessing cultures, valuing diversity, and managing the dynamics of difference). However, few students included the other elements of adapting to diversity (12%) and institutionalizing cultural knowledge (2%). Additionally, the majority of responses suggested that the students saw culture as being one-directional (i.e., something “others” have or that one culture is “correct”; e.g., “Be able to educate patients and help them overcome those barriers when cultural aspects are hindering health goals”), singular (i.e., people possess one culture rather than understanding culture can be multidimensional and intersectional; e.g., “They acknowledge when they don’t know what is culturally acceptable, or enough about that culture, and listen to the person and work to respect and acknowledge the cultures they do know of others.”), or monolithic (i.e., people who identify as being part of a given cultural group will have similar wants, needs, and lived experiences; e.g., “…[U]nderstanding what is important or valued in different cultures and understanding holidays and religious duties”). Coding these same responses to this open-ended question based on the Continuum of Cultural Proficiency [16, 18, 59] (Fig. 1) revealed that most students (76%) had a personal definition that suggested cultural precompetence; only 3% of definitions were coded as reaching cultural proficiency while 2% indicated cultural blindness or incapacity (Fig. 5). These definitions supplied by students demonstrated similar levels of understanding regardless of rotational status (*p =* 0.23).

### Graduate health student’ implicit associations

Regardless of rotation status, most IAT responses showed stronger automatic preferences towards the group often associated with more power and privilege in the United States [60–62] (Fig. 2C-D). For example, 68% of students had IAT results that indicated a slight to strong preference (values of 1–3) for White People over Black People, and 80% had results that showed a slight to strong preference for Abled Persons over Disabled Persons. However, the average values for the race and ability IATs were higher amongst participants who had completed one or more clinical rotations compared to those who had not yet started a rotation (*p =* 0.03 and *p =* 0.01, respectively).

## Discussion

There is wide recognition of the need to graduate emerging healthcare practitioners who possess highly specialized disciplinary knowledge and are also empathetic, effective communicators who can work within a DEIJ framework to address health disparities [13, 63–68]. However, there is not yet strong consensus on the most effective ways to accomplish these outcomes. Data from the present study corroborate and expand on prior findings, which have found that, while many students receive CP training, their experiences with the training can vary considerably [69–74]. Additionally, the data from the present study makes progress towards understanding, with more granularity, students’ beliefs, knowledge, experience, and perspectives related to their CP training and interests. Together, these findings catalog CP-related training experiences, which contextualize and provide insight into several implications for the teaching and learning of CP topics, as outlined further below.

### Variability in CP training experiences may be a function of the teaching and mentorship practices employed

In this study, as in others, responses indicate that student ratings and takeaways from CP training can vary considerably [69–74]. For example, while some students stated that their training was enlightening, other responses implied that it was not helpful. Furthermore, the plurality of students didn’t identify aspects of their training that they found particularly interesting or “eye opening.” This may have occurred if participants found this survey question too vague. Still, it may also speak to limitations of the trainings themselves, including the scope and integration (or lack thereof) in the curriculum content, structure and format of instruction, and modeling and confidence of instructors.

The present study confirms that while CP topics are often included in programs’ curricula; the amount of training can vary. This content may be relegated to distinct CP courses rather than being taught alongside, and integrated with, disciplinary content areas, which can minimize the impact [70, 71]. In addition to when and where CP is introduced, literature has suggested that the teaching methods used can influence the learning outcomes [74, 75]. Educators typically utilize instructional strategies such as those identified in the present study (e.g., lectures, discussions, and readings); however, in their review article, Brottman et al. found that lectures may be less effective than other instructional strategies [75]. Nevertheless, they note that the impact can be increased by combining lectures with other methods, such as role-playing or discussions [75]. While this analysis also suggested that immersive experiences, such as community-based education and clinical rotations, may be highly impactful [75], other studies have found that clinical experiences alone may not prepare students to provide culturally proficient care or shift beliefs and attitudes [69, 71, 72, 76]. The latter may be particularly true if trainees witness behaviors from formal or informal educators (e.g., faculty, attendings, residents, etc.) that do not model CP or do not emphasize its value in healthcare [45, 63]. Results from the present study indicated that while many students understood the importance of CP to both clinical and educational practices (at rates similar to those seen in others throughout healthcare [73, 77, 78]), many respondents estimated that they observed CP modeled strongly in the clinic or classroom by peers, faculty, and staff once a week on average. This rate may have important implications for how students integrate information across their learning experiences. For example, students may encounter messaging from instructors about how important CP is (or come into the training with this understanding), but if they don’t observe their mentors frequently applying those skills in professional practice, these two inputs may conflict with one another [45, 63, 74, 79].

Additional factors impacting the students’ perceptions of CP training may include the lessons’ content (and whether instruction on that topic has been received before) as well as their educators’ comfort with teaching topics related to CP and DEIJ. Previous literature suggests that schools and programs often emphasize certain content areas over others - doctor-patient relationships, socioeconomic status, and racism are often highlighted, while language and access issues in healthcare may be under-represented in trainings [70, 73]. While the reason for this may vary, studies have indicated that most faculty teaching CP topics are interested in them but do not have extensive training on the subject [74, 75, 80]. Therefore, they may feel less prepared to deliver this content compared to topics perceived as more scientific or clinical [74, 75, 80]. Clinical skills must incorporate CP, communication, and empathy rather than being supplemented by these, so it’s essential to continue to explore how students receive this training and how it informs their perspectives. Toward this end, it is important to consider efforts to “train the trainers.” While the current study focused on students’ experiences, it is nevertheless critical to recognize the role faculty play in fostering equitable and inclusive learning environments and modeling CP in their interactions with students, patients, and colleagues in the clinic and on campus. Ensuring that clinical and didactic faculty integrate CP training holistically into their curricula is also essential. This should be done in a way that utilizes not only evidence-based medicine but also evidence-based pedagogical principles.

### Students’ self-ratings and implicit biases may change throughout their training

The data showed how, overall, students frequently rated themselves as competent on three CP meta-skills and indicated interests and values related to ethnocultural empathy. However, those students currently engaged in clinical rotations largely regarded their skills associated with understanding cultural differences and their impacts on healthcare less highly than students who had not yet begun a rotation and those who had completed a rotation. As the current study did not conduct analyses longitudinally, it is unknown how individual students’ self-ratings would change throughout a training program. However, this finding between groups in the study’s population might indicate that students feel more confident in their knowledge when it is being used in the context of classroom discussions around theoretical situations or once they can look back on their clinical rotation from a new perspective, but feel less confident when they are in the midst of a clinical encounter. This suggestion is supported by the results of multiple studies which have found that, despite receiving training, graduates in health science disciplines often feel under-prepared to provide elements of inclusive and empathic care to patients with backgrounds different from their own [71, 73].

Interestingly, the data did not indicate an association between the amount of training a student received or the value they ascribed to the training and their desire for continued education on CP. Instead, students overwhelmingly indicated they would benefit from additional CP training, and many identified specific topics of interest. While it is possible that these particular concepts were not included in the student’s previous training, many of these, including those related to care decisions, race and ethnicity, and communicating with patients are likely to be part of curricula in the health sciences given their relevancy for healthcare careers. Thus, it is possible that this finding may be indicative of students’ commitment to continual learning and growth that is inherent in the CP framework and expected within the model of continuing education in health sciences. In fact, students’ responses to the question that asked them to indicate their personal definitions of CP often alluded to this continuous learning journey. However, many of the responses also reflected the idea that by learning about aspects of a particular cultural group, a student would know how to interact with anyone with that identity. This suggests a lack of understanding of intersectionality and how individuals’ lived experiences, needs, and goals are entirely their own. Furthermore, responses to this same question typically centered around the need to treat people with kindness and respect. This is inarguably important but doesn’t necessarily take the next step along the continuum toward cultural proficiency. This would involve not only being nice to others, but intentionally creating inclusive environments and advocating for changes that promote equity and serve the needs of all people. To this end, it’s crucial that CP training is designed with such learning objectives in mind, is holistic, and helps students practice working as allies and advocates to address systemic discrimination across systems, including healthcare [16].

As difficult as it can be for training to lead to long-term changes in conscious behaviors and beliefs, it can be even more challenging to address implicit associations and biases [20, 81–83]. Prior literature has indicated that emerging and practicing healthcare providers have implicit reactions that largely mirror those of the general public [1, 7, 84–88]. While these instantaneous thoughts do not necessarily result in negative actions, prior literature has found associations between IAT results and changes in care decisions, treatment planning, and patients feeling unheard or uncomfortable [1, 7, 89–92]. In the present study, the results from two different IATs showed a tendency to demonstrate an automatic preference for the group associated with more power in the United States [60–62]. However, there was a shift towards more neutral associations in groups that had completed rotations. This might suggest that continued training and service in diverse communities, along with introspection and reflection, can lead to changes in individual implicit associations. However, additional studies would be needed to examine whether these effects persist over time [20, 83].

### Limitations and future directions

While the data in this study make progress toward understanding CP education in the health sciences, there are limitations of this work that present opportunities for future research. First, these data were collected from student volunteers at a single institution, which has implications for the generalizability of the findings. Therefore, opportunities remain for future studies to capture a broader range of student perspectives across the institution’s programs and locations and to use both similar and expanded methodologies. For example, the use of survey-based instruments in the present study allowed for a significant study population and a feasible time investment from the participant; however, this comes with the limitation that it did not allow for follow-up questions to be asked or deeper responses to be explored. Therefore, future research might leverage additional methodologies like focus groups or interviews to allow for several outcomes. Namely, a more interactive data collection method would enable respondents to better expand on the subjective nature of some of the responses which could, in turn, increase the robustness of the qualitative themes that emerged from the data set. These formats could also help to ensure students understood the intended meaning of the questions being asked and to gain more context for some of the answers. In future work, for instance, there is an opportunity to explore sub-questions presented by the current data, such as: How integrated do students feel their CP training has been in their training? How effective and engaging do they feel specific learning activities and formats have been? How have the group discussions been structured? How does training vary from class to class or discipline to discipline? Regarding the latter, while the participant population in the present study was not sufficiently large or diverse enough to allow for robust sub-analyses to be run, in a larger sample, it would be both meaningful and informative to disaggregate the data to explore the experiences and perspectives of students from across identity groups, programs of study, and based on where students conducted their rotations and training. Future research also has an opportunity to explore CP training and experiences more broadly across healthcare education and in various instructional settings (allopathic and osteopathic).

Another valuable opportunity for future research is to expand upon these findings by observing this topic from several different vantage points. In the current study, the ability to interpret some of the data is limited by the fact that only student perspectives were collected. However, future studies could triangulate the results further by analyzing the students’ perspectives along with those of their instructors and by examining the curricula and instructional materials. This could provide valuable insight into the models and theories used to teach CP and serve to better contextualize the classroom experiences. Related limitations of this study are that the data were collected at a single time and did not measure the short- or long-term impacts of any particular learning intervention. As such, it is unclear how students’ perspectives, experiences, and self-ratings evolve over time and what the efficacy and retention of the trainings were. Therefore, future work could include capturing longitudinal data through repeated testing and through a pre/post structure.

Lastly, the instruments used in this study largely captured self-perception data and the structure did not mirror real-life situations. However, future research has the opportunity to further explore students’ feelings and values (including using additional validated instruments specific to healthcare, such as the Jefferson Scale of Empathy [93]) alongside real-time or authentic interpersonal interactions by using patient feedback surveys, observations of clinical behaviors, or assessments through clinical vignettes.

## Conclusions

The results of this study provide insight into the experiences of graduate students at an osteopathic institution related to training in CP. The data also indicate ways in which didactic and immersive clinical training have provided time and space for students to explore this critical topic and the learning activities they have engaged in to do so. The results further suggest that while students value empathy and CP in theory, there are additional opportunities to further consider the pedagogical approaches utilized to develop students’ CP knowledge, skills, and attitudes and how the learning objectives, content, and assessments are integrated within and across classrooms and clinical rotations. Together these data make progress towards understanding how CP is taught across health disciplines and informing how educators might leverage intentional, evidence-based pedagogical practices to contribute to the training and advancement of culturally proficient practitioners and citizens.

### Supplementary Information

**Additional file 1.**

**Additional file 2.**

## Data Availability

The data and materials used and analyzed as part of this study can be made available upon reasonable request to the corresponding author.

## Abbreviations

CP: Cultural Proficiency
CHC: Community Health Clinic(s) or Community Health Centers
DEIJ: Diversity, Equity, Inclusion, and Justice
IAT: Implicit Association Test(s)
EEI: ethnocultural empathy inventory
