# Supplementary material for: Examining the pedagogical practices that support cultural proficiency development in graduate health science students
Source: BMC Med Educ. 2024 Feb 9;24:130. doi: 10.1186/s12909-024-05097-8 (PMC10858479; doi:10.1186/s12909-024-05097-8)
Supplement: Supplementary file 1 — Additional file 1. [file 12909_2024_5097_MOESM1_ESM.pdf]

Additional File 1. Map and list of clinical site locations reported by study participants

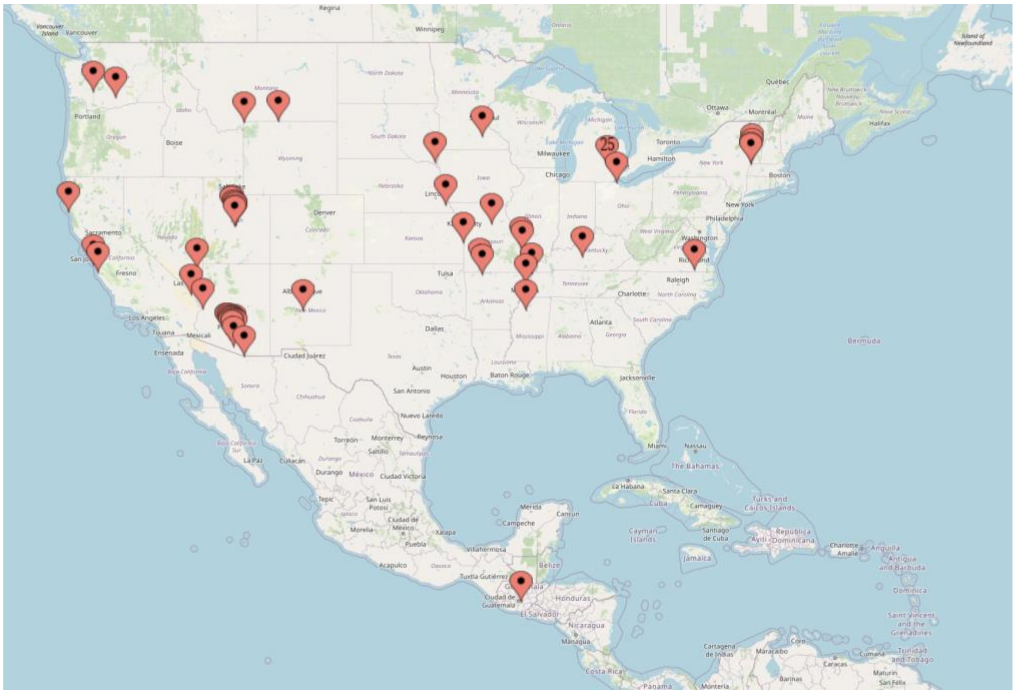

Map created with https://www.mapcustomizer.com/#

Ahwatukee, AZ
Albuquerque, NM
American Fork, UT
Arcata, CA
Auburn, WA
Bernie, MO
Billings, MT
Bolivar, MO
Bozeman, MT
Brandon, VT
Caliente, NV
Cape Girardeau, MO
Casa Grande, AZ
Castleton, VT
Chandler, AZ
Deer Valley, AZ
Ellensburg, WA
Florissant, MO
Fountain Hills, AZ
Gilbert, AZ
Glendale, AZ
Guatemala
Henderson, NV
Kansas City, KS
Kirksville, MO
Kingman, AZ
Louisville, KY
Memphis, TN
Mesa, AZ
Mesa, AZ
Middlebury, VT
Minneapolis, MN
Murray, UT
Oakland, CA
Omaha, NB
Orem, UT
Paradise Valley, AZ
Payson, UT
Phoenix, AZ
Poultney, VT
Provo, UT
Queen Creek, AZ
Richmond, VA
Saginaw, MI
San Jose, CA
Scottsdale, AZ
Sioux Falls, SD
Southfield, MI
Spanish Fork, UT
Springfield, MO
St. Louis, MO
Sun City West, AZ
Sun Lakes, AZ
Surprise, AZ
Tempe, AZ
Tucson, AZ
West Jordan, UT
