# Supplementary material for: Examining the pedagogical practices that support cultural proficiency development in graduate health science students
Source: BMC Med Educ. 2024 Feb 9;24:130. doi: 10.1186/s12909-024-05097-8 (PMC10858479; doi:10.1186/s12909-024-05097-8)
Supplement: Supplementary file 2 — Additional file 2. [file 12909_2024_5097_MOESM2_ESM.pdf]

Additional File 2. Questions and instruments used in the research survey included semi-quantitative and qualitative questions as well as the Ethnocultural Empathy Inventory.

Semi-Quantitative and Qualitative Questions

1. How important do you think it is for health care professionals to exhibit cultural proficiency in their clinical practice? (Likert-Type Question, 1-5 scale)
2. How important do you think it is for health care educators to exhibit cultural proficiency in their teaching practice? (Likert-Type Question, 1-5 scale)
3. What does it mean to you for someone to exhibit cultural proficiency? (Open-ended question)
4. Have you received training in cultural awareness, bias mitigation, and/or cultural proficiency? (Yes or No Question)
    a. If Q4 Yes: Please estimate the number of hours of cultural awareness, bias mitigation, and/or cultural proficiency training (e.g., online, classroom, etc.) that you have received during your tenure as a student at ATSU: (Open-ended question)
    b. If Q4 Yes: How has instruction and practice in cultural proficiency been incorporated into your training (select all that apply)? (Multiple Choices: Lectures, Readings, Demonstrations, Discussions, Reflections, Other, None)
    c. If Q4 Yes: Please rate how effective you think the training was in preparing you for a clinical rotation or your future career. (Likert-Type Question, 1-5 scale)
    d. If Q4 Yes: Have you received training in cultural proficiency outside ATSU (ex. in your undergraduate program, through a professional organization, etc.)? (Yes, No, Unknown)
        i. If Q4d Yes: Please estimate the number of hours of cultural awareness, bias mitigation, and/or cultural proficiency training (e.g., online, classroom, etc.) that you have received outside of ATSU: (Open-ended question)
5. In your experiences in the classroom or the clinic how frequently do you witness others (peers, faculty, and staff) demonstrating strong cultural proficiency? (Likert-Type Question, 1-5 scale)
6. Have there been particular experiences in your time as a student at ATSU that have been “eye-opening” or helpful in learning skills related to appreciating differences and mitigating bias? If so, please briefly describe the experience and how it impacted you? (Open-ended question)
7. Do you think you would benefit from additional training in cultural proficiency? (Yes, No, Uncertain)
8. What topics related to cultural proficiency would you like to learn more about? (Open-ended question)
9. How would you rate your understanding of the complex elements inherent to cultural differences and their impact on health and healthcare delivery? (Likert-Type Question, 1-5 scale)
10. How would you rate your ability to apply an understanding of cultural differences through active participation in diverse cultural experiences and opportunities? (Likert-Type Question, 1-5 scale)
11. How would you rate your ability to mitigate differences by communicating and acting in a supportive manner and recognizing other cultural group perspectives? (Likert-Type Question, 1-5 scale)

Ethnocultural Empathy Inventory [1,2]

1. When dealing with people of a different ethnicity or culture, understanding their viewpoint is a top priority for me.
2. It is easy for me to understand what it would feel like to be a person from a different culture.
3. I feel offended when I hear people make jokes about or use slang words to describe people from other ethnic backgrounds or cultures.
4. *I rarely think about the impact of an ethnic joke on people who are targeted.
5. I feel sorry for people of other ethnicities or cultures if I think they are being taken advantage of.

6. I share the anger of those who face injustice because of ethnic or cultural differences.
7. *It is difficult for me to put myself in the shoes of someone from another culture.
8. When making a group decision, I think that considering each person's perspective is more important than making a decision that's completely fair and impartial.
9. *I feel irritated when people of different ethnic or cultural backgrounds speak their native language around me.
10. *I feel impatient when communicating with people of different ethnicities or cultures than mine, regardless of how well they can communicate.
11. *I think the best decisions are made when we can remove any personal concerns, because emotions lead to biased decisions.
12. *I try to act based on the truth of a situation, not what others might want to believe or wish were true.
13. Making sure that everyone gets along in my team is one of my priorities.
14. *I try to look for a logical explanation or solution to almost every problem I encounter.
15. *I don't understand why people of different ethnicities or cultures feel they have to cling to their own values and traditions.

*Reverse scoring for these questions

[1] Wang Y-W, Davidson MM, Yakushko OF, Savoy HB, Tan JA, Bleier JK. The Scale of Ethnocultural Empathy: Development, validation, and reliability. J Couns Psychol 2003;50:221–34. https://doi.org/10.1037/0022-0167.50.2.221.

[2] Ross KG, Thornson CA, McDonald DP, Arrastia MC. The Development of the CCCI : The Cross-Cultural Competence Inventory. 7th Bienn Equal Oppor Divers Cult Symp 2009:77–112.
